# Sex Differences and Cytokine Profiles among Patients Hospitalized for COVID-19 and during Their Recovery: The Predominance of Adhesion Molecules in Females and Oxidative Stress in Males

**DOI:** 10.3390/vaccines11101560

**Published:** 2023-10-03

**Authors:** Olivera Mitrović-Ajtić, Dragoslava Đikić, Tijana Subotički, Sandra Bižić-Radulović, Bojana Beleslin-Čokić, Teodora Dragojević, Emilija Živković, Sanja Miljatović, Milica Vukotić, Dejana Stanisavljević, Juan Santibanez, Vladan P. Čokić

**Affiliations:** 1 Institute for Medical Research, University of Belgrade, National Institute of Republic of Serbia, Dr. Subotica starijeg 4, 11129 Belgrade, Serbia; oliveram@imi.bg.ac.rs (O.M.-A.); dragoslava@imi.bg.ac.rs (D.Đ.); tijana@imi.bg.ac.rs (T.S.); teodora.dragojevic@imi.bg.ac.rs (T.D.); emilija.zivkovic@imi.bg.ac.rs (E.Ž.); milica.tosic@imi.bg.ac.rs (M.V.); jfsantibanez@imi.bg.ac.rs (J.S.); 2 Clinic of Hematology, University Clinical Center of Serbia, Dr. Koste Todorovica 2, 11000 Belgrade, Serbia; bizics@yahoo.com; 3 Clinic of Endocrinology, Diabetes and Metabolic Diseases, University Clinical Center of Serbia, Dr. Subotica starijeg 13, 11000 Belgrade, Serbia; bojanabbc06@yahoo.com; 4 Clinic for Infectious and Tropical Diseases, University Clinical Center of Serbia, Bulevar oslobođenja 16, 11000 Belgrade, Serbia; 5 Institute for Medical Statistics and Informatics, Faculty of Medicine, University of Belgrade, 11000 Belgrade, Serbia; dejana.stanisavljevic@med.bg.ac.rs; 6 Centro Integrativo de Biología y Química Aplicada (CIBQA), Universidad Bernardo O’Higgins, Santiago 8370854, Chile

**Keywords:** COVID-19, sex hormones, vaccine, oxidative stress, cytokines

## Abstract

The severity and mortality of coronavirus disease 2019 (COVID-19) are greater in males than in females, though the infection rate is the same in the two sexes. We investigated sex hormone differences associated with the hyperinflammatory immune response to SARS-CoV-2 on the basis of patients’ cytokine profiles and vaccination statuses. Clinical and laboratory data of 117 patients with COVID-19 were collected to examine sex differences associated with oxidative stress markers, neutrophil extracellular traps (NETs), and plasma cytokine levels up to 5 months from hospital admission. The testosterone and free testosterone levels were low in male patients with COVID-19 and returned to normal values after recovery from the disease. The dihydrotestosterone (DHT) levels were transiently reduced, while the sex hormone-binding globulin levels were decreased in post-COVID-19 male patients. The levels of the inflammatory cytokines interleukin-6 (IL-6) and IL-10 appeared generally increased at diagnosis and decreased in post-COVID-19 patients. In females, the concentration of tumor necrosis factor-alpha was increased by four times at diagnosis. The levels of the coagulation markers intercellular adhesion molecule-1 (ICAM-1) and E-selectin were consistently upregulated in post-COVID-19 female patients, in contrast to those of vascular cell adhesion molecule-1 (VCAM-1), P-selectin, and chemokine IL-8. DHT increased the levels of reactive oxygen species in the neutrophils of male patients, while estradiol decreased them in females. Markers for NET, such as circulating DNA and myeloperoxidase, were significantly more abundant in the patients’ plasma. Sex hormones have a potential protective role during SARS-CoV-2 infection, which is weakened by impaired testosterone synthesis in men.

## 1. Introduction

Coronavirus disease 2019 (COVID-19) stimulates a hyperinflammatory response during which reactive oxygen species (ROS) induce chemokines responsible for the recruitment of inflammatory cells to the site of infection [1]. COVID-19 patients in intensive care units (ICU) present increased ROS levels and protein oxidation compared to non-ICU patients [2]. ROS induce the formation of neutrophil extracellular traps (NETs), whose levels are augmented in the plasma of COVID-19 patients and are correlated with inflammatory cytokine secretion and endothelial damage [3,4]. Furthermore, the concentration of endothelial cell adhesion molecules (such as vascular cell adhesion molecule-1 (VCAM-1) and intercellular adhesion molecule-1 (ICAM-1) is elevated in patients with COVID-19 and contributes to coagulation dysfunction [5].

Regarding the hyperinflammatory response, elevated levels of pro-inflammatory interleukin-6 (IL-6) in COVID-19 patients have been associated with increased patient mortality, ICU admissions, and ventilator requirements [6]. Higher levels of circulating IL-6, IL-10, IL-8, and tumor necrosis factor-alpha (TNF-α) were detected in non-survivors compared to survivors as well as in severe or critical COVID-19 cases [7,8]. The serum levels of interferon-gamma (IFN-γ), tumor growth factor-beta (TGF-β), and IL-8 are significantly higher in patients with COVID-19 [9]. Chemokines may be a direct cause of acute respiratory disease syndrome, the main complication leading to death in severe COVID-19 cases [10].

A poor T-cell response negatively correlates with aging and is associated with inferior clinical outcomes in male patients with COVID-19 [11]. A higher number of COVID-19-associated deaths have been reported for male patients compared to female patients, with a total mortality rate between 59% and 75% [12]. Low levels of total and free testosterone predict a poor prognosis and mortality in patients with COVID-19 admitted to ICU [13]. Semen analyses revealed decreased sperm motility and a higher number of immobile sperm in the first 3 months following COVID-19 infection [14].

Considering the sex-related differences in the clinical outcomes of COVID-19, we hypothesized that sex hormones have a protective role in the hyperinflammatory immune response to severe acute respiratory syndrome coronavirus 2 (SARS-CoV-2) infection and the consequent thrombosis, which is a major feature of COVID-19. We examined sex-related differences in the profiles of hormones and cytokines responsible for inflammation, chemotaxis, and coagulation (adhesion molecules) in patients with COVID-19 at diagnosis and up to 5 months after hospital admission and compared them with healthy donors. Besides a correlation with biochemical parameters, clinical outcome, comorbidities, and vaccination status, we also observed the influence of sex hormones on ROS-induced formation of NETs. We demonstrated sex-related differences in the inflammatory response and in the production of coagulation factors in COVID-19 patients.

## 2. Materials and Methods

### 2.1. Sample Collection

Peripheral blood samples from healthy donors and patients with COVID-19, confirmed by positive RT-PCR testing, were collected at the Clinic for Infectious and Tropical Diseases, University Clinical Centre of Serbia, Belgrade, Serbia. From the same post-COVID-19 patients, blood was collected 2.5 (males, 74.9 ± 18.8 days, females, 67.7 ± 28 days) and 5 months (males, 155 ± 36.4 days, females, 151.7 ± 37.4 days) after hospital admission (the average age at diagnosis was 54.5 ± 14.4 years for the 77 male patients examined and 61.8 ± 13.1 years for the 40 female patients). We observed 37 healthy donors (20 females and 17 males). In our cohort, 28% of the COVID-19 male patients and 35% of the female patients were overweight. The levels of inflammatory cytokines appear to be an independent risk factor even after adjusting for age and considerably influence the outcomes of COVID-19 [7,12]. Information on patients’ sociodemographic and clinical characteristics was extracted from their medical records, including vaccination status (categorized as 0—none vaccinated; 1—one dose; 2—two doses of vaccine; and 3—booster), time since disease diagnosis, severity of the clinical outcomes (ranked as 1—mild; 2—moderate; 3—severe; 4—critical; and 5—deceased) and comorbidities at diagnosis (ranked according to the number of accompanying disorders). The study was conducted in accordance with the Declaration of Helsinki and approved by the Ethics Committee of the University Clinical Centre of Serbia (protocol code 570/12) and the Institute for Medical Research (131/2020). This study included 117 patients with COVID-19 examined between March 2021 and April 2022. The patients were diagnosed according to the Clinical Management of COVID-19 guidelines by the World Health Organization. All patients signed an informed consent approved by the institutional review board. For the analysis of a cytokine panel, 20 mL of peripheral blood was collected from the COVID-19 patients and placed in a plasma separator tube (BD Vacutainer K2E, EDTA). All samples were kept at room temperature for 20 min prior to centrifugation for 15 min at 2000 RPM. Plasma aliquots were stored at −80 °C and analyzed within ten days. In our study, the male patients’ sample size was larger compared to the female patients’, reflecting the fact that the majority of hospitalized patients with COVID-19 are males. Indeed, the number of female patients with COVID-19 was two times lower in our hospital, supporting the observation that females are less affected than males.

### 2.2. ELISA Assay

Plasma samples obtained from the healthy controls and COVID-19 patients at diagnosis, 2.5 and 5 months after disease diagnosis (Table S1), were analyzed to assess the levels of dihydrotestosterone (DHT), estradiol, sex hormone-binding globulin (SHBG), IL-6, IL-10, IL-1β, IL-8, IFN-γ, TGF-β, TNF-α, monocyte chemoattractant protein-1 (MCP-1), ICAM-1, VCAM-1, P-selectin, and E-selectin in plasma using ELISA kits (Elabsciences Biotechnology Inc., Houston, TX, USA, Table S2), according to the manufacturer’s instructions; no significant cross-reactivity or interference with analogues was observed. Testosterone normal plasma levels in men: 8.64–29 nmol/L in <50-year-olds, 6.68–25.7 nmol/L in >50-year-olds (19.5 ± 9.1 nmol/L, *n* = 5 healthy donors); normal levels in women: 0.29–1.67 nmol/L in <50-year-olds, 0.101–1.42 nmol/L in >50-year-olds (1.1 ± 0.4 nmol/L, *n* = 5 healthy donors). Free testosterone normal plasma levels in men: 15–50 pg/mL (29.8 ± 12.3 pg/mL, *n* = 4); normal levels in women: <4.2 pg/mL (2.1 ± 0.6 pg/mL, *n* = 5). They were commercially obtained using Cobas 6000 (Roche Diagnostics International AG, Rotkreuz, Switzerland) and Maglumi 1000 (Snibe Diagnostic, Shenzhen, China), respectively. The normal range of plasma DHT levels is between 112 and 955 pg/mL [15]. All plasma samples were tested in duplicate, and the results were obtained from a standard curve that was created using recombinant standards and expressed as the average concentration of each tested cytokine in adequate units. The measurements were performed on an ELISA Multiscan Plus plate reader (Labsystems, Vantaa, Finland).

### 2.3. NETs Quantity from the Plasma of the Patients with COVID-19

For the NETs study, blood samples from 44 COVID-19 patients (35 males, 9 females) and 9 healthy controls (5 males, 4 females) were collected in tubes treated with ethylenediaminetetraacetic acid (EDTA). The plasma was separated by centrifugation and used to analyze the NET markers, i.e., the concentration of circulating DNA (Circulating DNA Quantification Kit, Abcam, Cambridge, UK) and myeloperoxidase activity (MPO, Myeloperoxidase Activity Assay Kit, Elabsciences Biotechnology Inc.). Fluorescence was measured using a Perkin Elmer Wallac 1420 Victor2 instrument (Lianstriant, United Kingdom), and absorbance was read using an ELISA microplate reader (RT-6100, Raito, Shenzhen, China).

### 2.4. Nitro Blue Tetrazolium (NBT) Test of ROS Activity

Neutrophils were isolated from 20 COVID-19 patients (13 males, 7 females) and 6 healthy controls (3 males, 3 females) after separating different cell fractions using Lymphocyte Separation Medium (LSM, Capricorn Scientific GmbH, Ebsdorfergrund, Germany) and performing a hypotonic lysis of the precipitated erythrocytes. Neutrophils were resuspended in RPMI-1640 medium (Capricorn Scientific GmbH) supplemented with 10% fetal calf serum (FCS, Sigma Aldrich, St. Louis, MO, USA), L-glutamine, and penicillin/streptomycin (Capricorn Scientific GmbH) and incubated at 37 °C/5% CO_2_. The isolated cells (4–5 × 10^6^ neutrophils per well) were plated in 24-well plates and treated with different concentrations (1 nM, 10 nM and 100 nM) of DHT (5α-androstain-17β-ol-3-one, Sigma Aldrich), β-estradiol (EST, Tocris, Bristol, UK; 1 nM, 10 nM, and 100 nM), or the androgen receptor blocker flutamide (Sigma Aldrich, 100 nM) for 30 min. NETs were stimulated with phorbol-12-myristate 13-acetate (PMA, Sigma Aldrich) at concentrations of 10 and 100 ng/mL. Then, 100 µL of the neutrophil suspension was plated into 96-well plates for the nitro blue tetrazolium (NBT) test. The NBT reagent (Sigma Aldrich, 5 mg/mL) was added to the wells. The neutrophils were incubated for 30 min at 37 °C in a humidified atmosphere. The reduction process was stopped by adding 100 µL of acidified 10% SDS/HCl (sodium dodecyl sulphate, Promega Corporation, Madison, WI, USA). The absorbance was read at 540 nm on an ELISA reader (RT-6100, Raito, Shenzhen, China). To define the influence of ROS on DNA, the neutrophils were treated for 2 h with DHT, estradiol, and flutamide. Cell suspensions were isolated from 11 COVID-19 patients (6 males, 5 females) and 6 healthy controls (3 males, 3 females) and used to prepare cytospin slides (2 × 10^4^ cells) by centrifugation (1000 rpm, RT, for 5 min). The slides were fixed in acetone. Before proceeding with the immunocytochemistry analysis, the microscope slides were washed with phosphate-buffered solution (PBS). Endogenous peroxidase was blocked with a 3% H_2_O_2_ solution for 10 min. The slides were incubated with a primary anti-histone H3 monoclonal antibody and an anti-MPO polyclonal antibody (Elabscience Biotechnology Inc.) at +4 °C overnight. After incubation with biotinized anti-rabbit immunoglobulins, the cells were treated with streptavidin conjugated with horseradish peroxidase (UltraVision Detection System, HRP, Thermo Scientific, London, UK). Finally, the slides were incubated in a solution of substrate–chromogen (Liquid DAB+Substrate Chromogen System, Dako, Glostrup, Denmark). Mayer’s hematoxylin was used for contrast. The slides were analyzed using an Olympus Provis AX70 microscope, Tokyo, Japan.

### 2.5. Statistical Analysis

The normality of data distribution was examined by the Shapiro–Wilk and Kolmogorov–Smirnov tests. Differences between groups were analyzed using the Student’s *t*-test. When the distribution was not normal, the Mann–Whitney test was used for intergroup comparisons. Differences between variables at diagnosis and after 2.5 or 5 months were tested by the Wilcoxon signed-rank test. The correlations between numerical variables were assessed by Pearson’s or Spearman’s correlation coefficients. All statistics were performed using Prism 6 software (GraphPad Software Inc., San Diego, CA, USA). The results are expressed as mean ± SEM. The level of significance was 5%.

## 3. Results

### 3.1. Levels of Sex Hormones in the COVID-19 and Post-COVID-19 Patients

**Figure 1:**
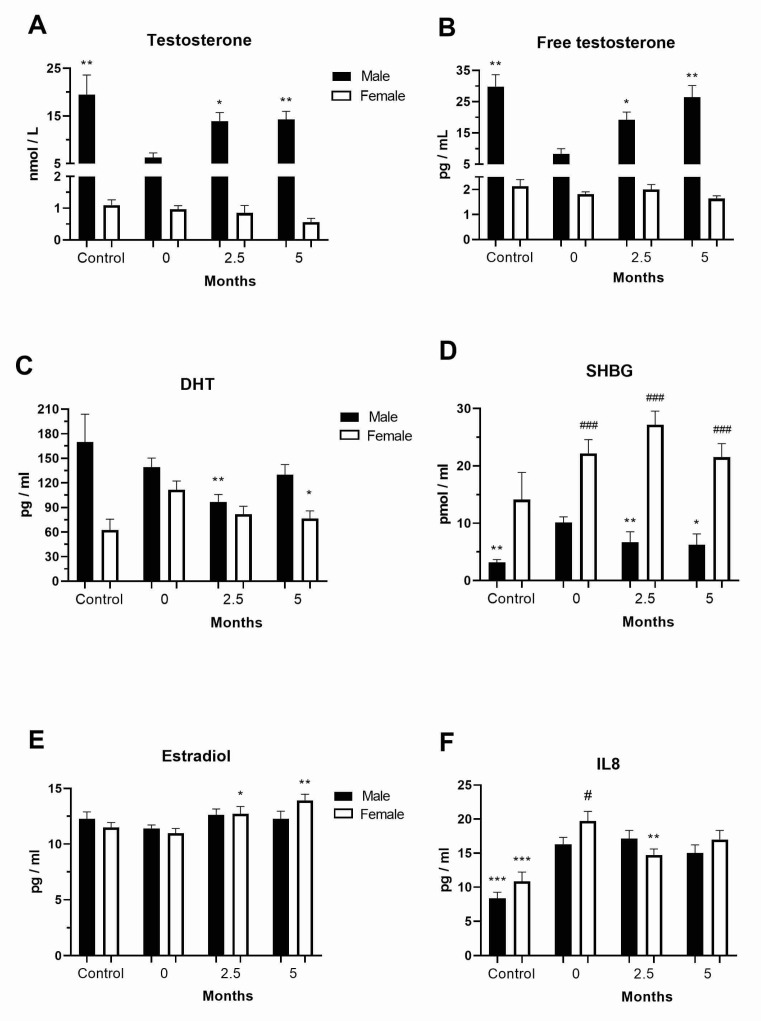
Sex hormone and chemokine levels in the peripheral blood of COVID-19 and post-COVID-19 patients. Levels in the peripheral blood plasma of healthy donors (control, *n* = 4–12 per sex) and COVID-19 female and male patients of (**A**) testosterone (*n* = 14), (**B**) free testosterone (*n* = 14), (**C**) DHT, (**D**) SHBG, (**E**) estradiol, and (**F**) IL-8 at diagnosis (0, *n* = 55–91) and 2.5 (*n* = 38–69) and 5 (*n* = 22–54) months after hospital admission (Table S1). Values are mean ± SEM. * *p* < 0.05, ** *p* < 0.01, *** *p* < 0.001 vs. same sex COVID-19 patients at diagnosis; ^#^ *p* < 0.05, ^###^ *p* < 0.001 vs. male pair.

To examine the role of sex-related differences in mortality and clinical outcomes in COVID-19 patients, we analyzed the levels of sex hormones in our COVID-19 patients at diagnosis as well as 2.5 and 5 months after hospital admission. The testosterone levels were below the normal values at hospital admission in the COVID-19 male patients (6.27 ± 2.58 nmol/L) but were normalized in the post-COVID-19 male patients (Figure 1A). The free testosterone levels were also below the normal values at hospital admission in the COVID-19 male patients (8.31 ± 4.44 pg/mL) but appeared to recover in the post-COVID-19 male patients (Figure 1B). The DHT levels were lower in the post-COVID-19 male (96.61 ± 66.48 pg/mL) and female (39.31 ± 101.16 pg/mL) patients (Figure 1C). The SHBG levels were increased at diagnosis and decreased in the post-COVID-19 male patients (*p* < 0.01, Figure 1D). The estradiol levels were stable in the COVID-19 female patients at hospital admission (10.96 ± 2.32 pg/mL) but increased after 5 months (13.9 ± 2.25 pg/mL, *p* < 0.01, Figure 1E).

### 3.2. Levels of Chemokines in the COVID-19 and Post-COVID-19 Patients

The level of the chemoattractant IL-8 was generally increased in the examined COVID-19 patients at diagnosis (males, 16.3 ± 7.8 pg/mL, females, 19.7 ± 7.5 pg/mL) and decreased in the post-COVID-19 female patients (*p* < 0.01, Figure 1F). The concentrations of MCP-1, a chemokine that regulates the migration and infiltration of monocytes/macrophages, were two-fold increased in COVID-19 female (*p* < 0.01) patients at diagnosis and further increased in the post-COVID-19 female patients after 2.5 months from hospital admission (850.47 ± 468.33 pg/mL, *p* < 0.05, Table S3). The levels of both IL-8 and MCP-1 were steady in the COVID-19 and post-COVID-19 patients.

### 3.3. Levels of Inflammatory Cytokines in the COVID-19 and Post-COVID-19 Patients

**Figure 2:**
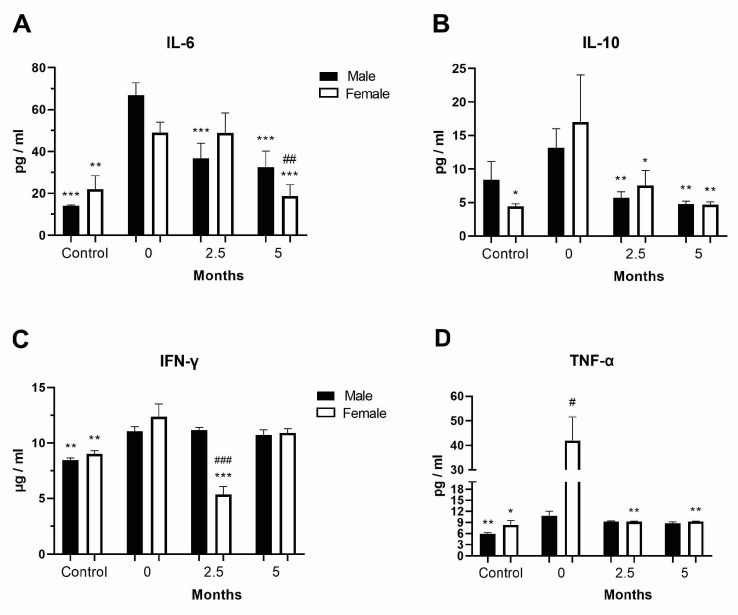
Inflammatory cytokine levels in the peripheral blood of COVID-19 and post-COVID-19 patients. Levels in the peripheral blood plasma of healthy donors (control, *n* = 7–11 per sex) and female and male COVID-19 patients of (**A**) IL-6, (**B**) IL-10, (**C**) IFN-γ, (**D**) TNF-α o at diagnosis (0, *n* = 47–78) and 2.5 (*n* = 41–67) and 5 (*n* = 22–42) months after hospital admission (Table S1). Values are mean ± SEM. * *p* < 0.05, ** *p* < 0.01, *** *p* < 0.01 vs. same sex COVID-19 patients at diagnosis; ^#^ *p* < 0.05, ^##^ *p* < 0.01, ^###^ *p* < 0.001 vs. male pair.

Considering that a hyperinflammatory response can occur in COVID-19 patients, we examined the levels of inflammatory cytokines in the COVID-19 patients at diagnosis as well as 2.5 and 5 months after hospital admission. The levels of the pro-inflammatory cytokine IL-6 were generally increased at diagnosis in the COVID-19 male patients, while they were decreased in the same patients at follow-up but were still elevated after 5 months compared to healthy controls (*p* = 0.049, Figure 2A). A similar trend was observed for the anti-inflammatory cytokine IL-10 (Figure 2B). IL-1β concentration was significantly increased only in the COVID-19 female patients at diagnosis (*p* < 0.05, Table S3). IFN-γ levels were generally increased in the COVID-19 patients at diagnosis (*p* < 0.01), whereas they were transiently reduced in the post-COVID-19 female patients 2.5 months after hospital admission (Figure 2C). The concentration of the anti-inflammatory cytokine TGF-β was decreased (*p* < 0.001) in the COVID-19 and post-COVID-19 patients compared to healthy controls (Table S3), while that of the pro-inflammatory cytokine TNF-α was increased by four times in the female COVID-19 patients compared to the male patients at diagnosis (Figure 2D). TNF-α levels returned to normal values as early as 2.5 months after hospital admission in post-COVID-19 female patients.

### 3.4. Levels of Cell Adhesion Molecules in the COVID-19 and Post-COVID-19 Patients

**Figure 3:**
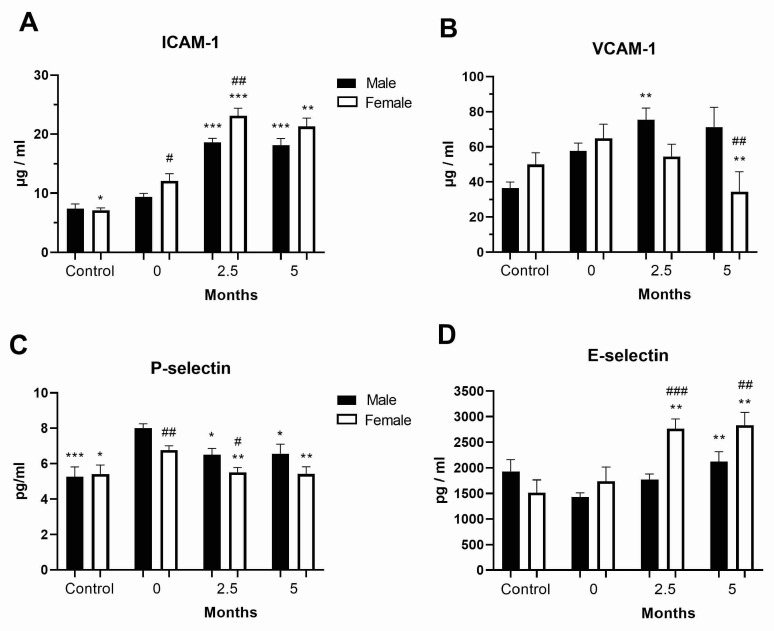
Levels of cell adhesion molecules in the peripheral blood of the COVID-19 and post-COVID-19 patients. Levels in the peripheral blood plasma of healthy donors (control, *n* = 4–14 per sex) and the COVID-19 female and male patients of (**A**) ICAM-1, (**B**) VCAM-1, (**C**) P-selectin and (**D**) E-selectin at diagnosis (0, *n* = 49–71) and 2.5 (*n* = 41–79) and 5 (*n* = 22–48) months after hospital admission (Table S1). Values are mean ± SEM. * *p* < 0.05, ** *p* < 0.01, *** *p* < 0.01 vs. same-sex COVID-19 patients at diagnosis; ^#^ *p* < 0.05, ^##^ *p* < 0.01, ^###^ *p* < 0.001 vs. corresponding male patients.

The level of the cell adhesion molecule ICAM-1 was increased in the female COVID-19 patients at diagnosis and was further generally increased in all COVID-19 patients at follow-up, though the increase was greater in females (*p* < 0.001, Figure 3A). VCAM-1 was upregulated in the post-COVID-19 male patients compared to the female ones (*p* < 0.01, Figure 3B). P-selectin level was also increased in the COVID-19 and post-COVID-19 male patients compared to the female ones (Figure 3C). In contrast, E-selectin was upregulated in the post-COVID-19 female patients compared to the male ones (Figure 3D). Concentration values for all sex hormones, cytokine/chemokines, and adhesion molecules are shown in Table S3.

### 3.5. Reactive Oxygen Species Activity in Neutrophils

**Figure 4:**
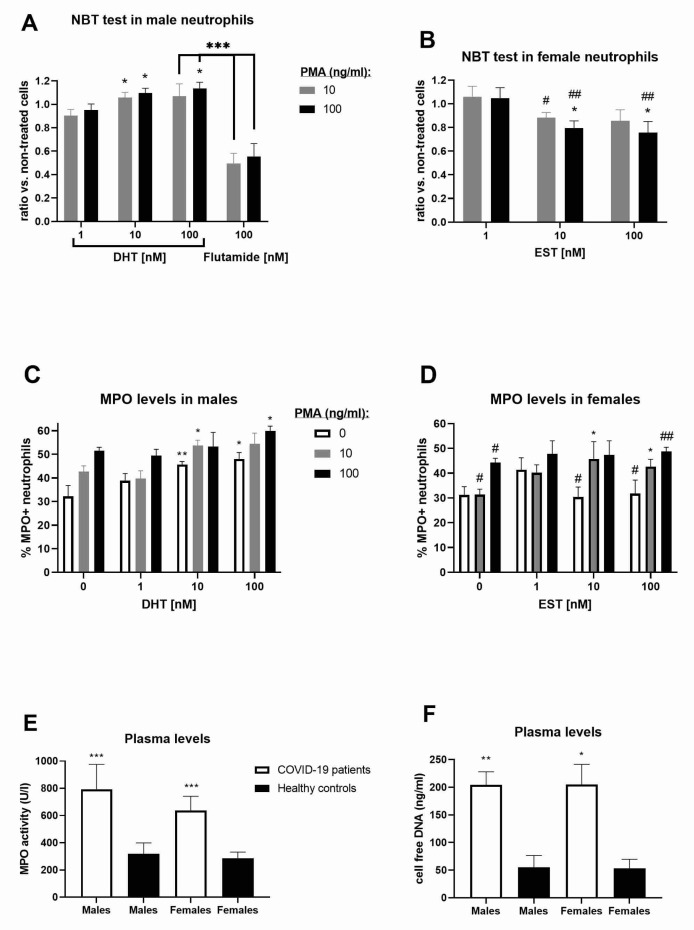
NETs in patients with COVID-19. Neutrophil activation by PMA was observed using the NBT test in neutrophils from (**A**) COVID-19 male patients during in vitro treatment with DHT and flutamide (*n* = 10–13) and (**B**) COVID-19 female patients during estradiol (EST) treatment (*n* = 6–7). NETs were determined based on neutrophils’ activation by PMA and the expression of MPO in (**C**) COVID-19 male patients during DHT treatment (*n* = 6) and (**D**) COVID-19 female patients during EST treatment (*n* = 5). NETs were quantified in the plasma of healthy controls (5 males and 4 females) and patients with COVID-19 by measuring (**E**) MPO activity and (**F**) circulating cell-free DNA in females (*n* = 9) and males (*n* = 35). Values are mean ± SEM. * *p* < 0.05, ** *p* < 0.01, *** *p* < 0.01 vs. controls (1 for graphs (**A**,**B**); 0 for graphs (**C**,**D**); and healthy controls for graphs (**E**,**F**)) or indicated columns; ^#^ *p* < 0.05, ^##^ *p* < 0.01 vs. COVID-19 male patients in corresponding panels (**A**,**C**).

Using the NBT assay, we measured the in vitro ROS activity by analyzing the activation of neutrophils from patients with COVID-19 in the presence of increasing concentrations of the androgen receptor agonist DHT (at the physiological concentration of 1 nM (290 pg/mL) or at a supraphysiological concentration ≥10 nM (2904 pg/mL)) or the antagonist flutamide (100 nM). A respiratory burst occurred in neutrophils, causing the release of ROS. Therefore, we performed an NBT test and demonstrated that, in COVID-19 male patients, DHT dose-dependently increased the levels of ROS in neutrophils (Figure 4A), while, in female patients, estradiol decreased them (Figure 4B). Moreover, ROS activity was decreased in female vs. male neutrophils during in vitro exposure to sex hormones (Figure 4B). As a selective androgen receptor antagonist, flutamide induced a two-fold reduction in ROS activity in neutrophils from male patients with COVID-19 (Figure 4A). DHT or estradiol did not significantly affect the in vitro ROS activity in neutrophils from healthy controls.

### 3.6. Quantification of NETs from the Plasma of Patients with COVID-19

We visualized NETs based on the expression of histone H3 and MPO evaluated by immunohistochemistry (Figure 4C,D) in patients with COVID-19. Only the highest concentration of DHT (100 nM, *p* < 0.05, from 35.3% to 49.8%) significantly increased the number of histone H3-positive neutrophils in the male patients with COVID-19; in contrast, estradiol (1, 10, 100 nM) had a weaker effect in the female patients with COVID-19 (up to 38%). Also, in the presence of the DHT blocker flutamide (100 nM), the number of H3-positive neutrophils remained at baseline (up to 36%) in the male patients with COVID-19. The difference in NETs was more obvious when examined using the anti-MPO antibody and was more consistent in the male patients with COVID-19 during DHT treatment (Figure 4C). The frequency of MPO-positive neutrophils was reduced in the COVID-19 female vs. the male patients and appeared to be sporadically under the influence of estradiol (Figure 4D). In addition, in the presence of flutamide, the number of MPO-positive neutrophils remained at the basal level in the male patients with COVID-19; in contrast, at the highest concentration of PMA (100 ng/mL), flutamide significantly reduced the number of MPO-positive neutrophils (from 51% to 37%, *p* = 0.019). We quantified the increased NETs in the plasma of the male patients with COVID-19 by measuring the circulating cell-free DNA (204.7 ± 138.1 vs. 55 ± 48.3 ng/mL in healthy controls) and MPO activity (793.3 ± 182.7 vs. 318.1 ± 80.3 U/L in healthy controls). We also quantified the increased NETs in the plasma of the female patients with COVID-19 in the same way by measuring the circulating cell-free DNA (205 ± 109.4 vs. 53.1 ± 33.3 ng/mL in healthy controls) and MPO activity (637.5 ± 104.6 vs. 286.9 ± 44.7 U/L in healthy controls). The levels of MPO activity and circulating cell-free DNA were generally increased in patients with COVID-19 (Figure 4E,F). After 2.5 months from hospital admission, MPO activity remained elevated in both post-COVID-19 male (233.5 ± 118.2 U/L, *n* = 14) and female (203.1 ± 77.1 U/L, *n* = 7) patients. The MPO levels were increased in neutrophils from the male patients with COVID-19 and stimulated by DHT, while markers of NETs were more abundant in the plasma from the COVID-19 and post-COVID-19 patients. The amount of neutrophils and other blood cells is presented in Table S4. The quantity of neutrophils is generally reduced in post-COVID-19 patients (*p* < 0.001) compared to COVID-19 patients (Table S4). In contrast, the quantity of lymphocytes (*p* < 0.001), monocytes (*p* < 0.01), and platelets (*p* < 0.001) were generally increased in post-COVID-19 patients (*p* < 0.001), compared to COVID-19 patients (Table S4).

### 3.7. Correlation of Biochemical Parameters with the Levels of Cell Adhesion Molecules and Sex Hormones in the COVID-19 and Post-COVID-19 Patients

Taking into account the vaccination status of the examined COVID-19 patients, we evaluated the existence of a correlation between biochemical parameters and the levels of the adhesion molecules and sex hormones considered. The biochemical parameters of COVID-19 and post-COVID-19 patients are shown in Table 1. INR was increased after 2.5 months of hospital admission, while other parameters were generally reduced in post-COVID-19 patients.

To describe the clinical significance of the observed levels of biochemical markers, cell adhesion molecules, and sex hormones, we analyzed and correlated them simultaneously in three ways, i.e., according to a biochemical profile, a time scale of their variations, and sex differences (Table 2). Testosterone was positively correlated with free testosterone at diagnosis in COVID-19 male patients (Spearman r = 0.9286, *p* = 0.0067). Free testosterone was in positive correlation with the number of comorbidities (Pearson r = 0.04, *p* = 0.831). Estradiol was positively correlated with INR (r = 0.634, *p* = 0.013), PTS (r = 0.659, *p* = 0.009), creatinine (r = 0.620, *p* = 0.016), CK (r = 0.636, *p* = 0.030), and urea (r = 0.672, *p* = 0.011) levels in post-COVID-19 female patients. AST (r = 0.569, *p* = 0.007), ALT (r = 0.573, *p* = 0.008), and GGT (r = 0.589, *p* = 0.023) were positively correlated with VCAM-1 expression in post-COVID-19 patients. LDH was negatively correlated with E-selectin (r = −0.875, *p* = 0.001) and VCAM-1 (r = −0.731, *p* = 0.030) expression in post-COVID-19 female patients. DHT was negatively correlated with D-dimer (r = −0.349, *p* = 0.013) and CRP (r = −0.283, *p* = 0.049) levels in post-COVID-19 patients. In contrast to SHBG, DHT was positively correlated with CK (r = 0.365, *p* = 0.018) in post-COVID-19 patients. Fibrinogen was negatively correlated with P-selectin (r = −0.627, *p* = 0. 005) and SHBG (r = −0.778, *p* = 0.011, Table 2) expression. Spearman’s correlation of biochemical parameters with the levels of inflammatory cytokines, chemokines, cell adhesion factors, and sex hormones in COVID-19 and post-COVID-19 patients is shown in Table S5.

### 3.8. Correlation of Vaccination and Biochemical Parameters with the Levels of Inflammatory Cytokines and Chemokines in COVID-19 and Post-COVID-19 Patients

Taking into account the vaccination status of the examined COVID-19 patients, we examined the existence of a correlation between biochemical parameters and the levels of cytokines, chemokines, and hormones considered. In our cohort, 39.3% of the male COVID-19 patients and 41.2% of the female COVID-19 patients had been vaccinated at diagnosis. In addition, 31.4% of the vaccinated males and 35.7% of the vaccinated females with COVID-19 had received a booster dose of the vaccine. IL-6 levels were significantly higher (Mann–Whitney test, *p* = 0.0132) in the vaccinated COVID-19 patients (75.1 ± 37 vs. 56.8 ± 50.9 pg/mL) at diagnosis. DHT (130.9 ± 80.5 vs. 74.8 ± 47.9 pg/mL, *p* = 0.005) and P-selectin (5.2 ± 1.3 vs. 7.4 ± 2.5 pg/mL, *p* = 0.024) levels were also significantly higher in the vaccinated post-COVID-19 male patients after 2.5 and 5 months of diagnosis, respectively. Finally, P-selectin levels were significantly higher in the vaccinated post-COVID-19 patients, both females (2618.2 ± 1116.6 vs. 1497.2 ± 892.6 pg/mL, *p* = 0.043) and males (2339.6 ± 1122.1 vs. 1804.6 ± 1068.6 pg/mL, *p* = 0.034), after 5 months of diagnosis. This higher level of IL-6 was not observed in the post-COVID-19 patients. To describe the clinical significance of the observed levels of inflammatory cytokines and chemokines, we analyzed and correlated them simultaneously in three ways, i.e., according to a biochemical and clinical profile, a time scale of their variations, and sex differences (Table 2 and Table S5). Fibrinogen showed a positive correlation with INF-γ (r = 0.588, *p* = 0.038) and MCP-1 (*p* = 0.027) in post-COVID-19 female patients. CRP was negatively correlated with IL-6 (r = −0.553, *p* = 0.011) and positively correlated with MCP-1 (r = 0.389, *p* = 0.019) in the post-COVID-19 patients. Creatinine was positively correlated with INF-γ (r = 0.535, *p* = 0.001) in the post-COVID-19 female patients but negatively correlated with IL-1β (r = −0.546, *p* = 0.006) in the post-COVID-19 male patients. Inversely to P-selectin and IFN-γ, vaccination was in positive correlation with E-selectin, ICAM-1, and VCAM-1 in post-COVID-19 male patients. DHT was in positive correlation with clinical outcome and comorbidities in COVID-19 female and male patients, respectively (Table 2 and Table S5).

## 4. Discussion

To address the role of sex differences in COVID-19 severity, we examined the levels of cytokines critical for inflammation, as well as chemotaxis and endothelial dysfunction responsible for clinical outcomes, in patients with COVID-19 at disease diagnosis and during a prolonged clinical follow-up. In humans, sexual dimorphism in the immune response has been well established, with females exhibiting lower infection rates than males for a variety of bacterial, viral, and parasitic pathogens [16]. Epidemiological data and observational reports from the previous SARS-CoV-1 epidemic and the most recent COVID-19 pandemic have a shared feature: disease severity and mortality are expected to be greater in men than in women [17]. Previous reports showed that the average COVID-19 male case fatality rate (CFR) in 38 countries is 1.7 times higher than the average COVID-19 female CFR [18]. The risk of death increases with age for both sexes, but at all ages above 30 years, men have a significantly higher risk of death than women [18]. The increased male mortality is consistent across age groups, with the highest CFR observed for middle-aged men (50–59 years), which is consistent with the average age (54.5 years) of the examined COVID-19 patients [19].

As a means of virus entry in the cells, the ACE2 receptor is downregulated by SARS-CoV-2. The highest gene and protein expression of ACE2 was observed in the intestinal tract, kidney, testis (Leydig and Sertoli cells), gallbladder, and heart, while it is minimal in the female reproductive organs [20]. The largest amount of testosterone (>95%) is produced by Leydig cells in the testes. Acute testicular changes have been observed in COVID-19 patients (reduced Leydig cells), with microthrombosis in the testicular vasculature, mild lymphocytic inflammation, and absent or minor SARS-CoV-2 presence [21]. Men with hypogonadism were found to be more likely than men with eugonadism to be hospitalized with COVID-19, while men receiving testosterone therapy had a similar risk of hospitalization as men with eugonadism [22].

In accordance with our presented results, the level of total testosterone in the serum is significantly lower in COVID-19 patients and even more decreased in patients with severe disease and in deceased patients [23,24]. Testosterone has anti-inflammatory and immunomodulatory protective effects, which are essential for the antiviral response in males, and testosterone deficiency is related to endothelial dysfunction and thrombocyte malfunction, predisposing to thromboembolic complications [15]. Therefore, the reduction of Leydig cells, as the main source of testosterone, was responsible for the low level of total testosterone and free testosterone and consequently reduced anti-inflammatory effects of testosterone in the hyperinflammatory response to the infection of COVID-19. With a concentration 7–10-fold lower than that of testosterone, the DHT levels were found to be reduced in COVID-19 male patients [25]. Observed plasma DHT levels were at or below minimal normal levels in both COVID-19 and post-COVID-19 patients. After recovery, the reduced DHT level in the first few months is a delayed reaction to the reduced testosterone level at diagnosis.

Testosterone synthesis is regulated by negative feedback to the hypothalamic–pituitary (follicle-stimulating hormone (FSH)/luteinizing hormone (LH))–testicular axis [26]. With a strong correlation with disease severity and mortality, male patients with COVID-19 had significantly low testosterone and SHBG levels and high levels of LH; in contrast, their levels of free testosterone and FSH were not affected, while they were lower in patients who required admission to the ICU [27,28]. The FSH and LH levels were found to be significantly increased in COVID-19 female patients [27]. In an additional study, FSH was also increased in COVID-19 male patients [13]. In males infected with SARS-CoV-2, the levels of most sex-related hormones (testosterone, FSH, and LH) were found within the normal reference ranges after 3 months of recovery from COVID-19 [29]. The study did not show the existence of any damage to the testicular parenchyma in post-COVID-19 patients [29]. Therefore, the recovery of testicular function is responsible for the normalized levels of testosterone and free testosterone in post-COVID-19 patients. We found higher plasma SHBG levels in COVID-19 male patients at diagnosis, which decreased during follow-up. Compared to those in males, the SHBG levels remained elevated in the COVID-19 and post-COVID-19 female patients. Age-dependent reference ranges for normal SHBG levels in men (30.83–50.92 pmol/mL) and women (40.88–78.32 pmol/mL) have been reported [30]. Observed SHBG levels were below reference values in both the COVID-19 and post-COVID-19 patients and reflected their reduced testosterone and DHT levels.

Total testosterone deficiency and low free testosterone levels are associated with prolonged hospitalization and elevated neutrophil-to-lymphocyte ratios (NLRs), high-sensitivity CRP, IL-6, and D-dimer [24,31]. We showed that the levels of CRP and D-dimer were negatively correlated with those of DHT. The levels of IFN-γ, IL-1Ra, IL-6, and MCP-1 were elevated in male patients with severe COVID-19, while those of TNF-α were high in COVID-19 female patients [25]. We showed a largely increased TNF-α concentration in COVID-19 female patients at diagnosis that was normalized in post-COVID-19 patients. Produced by innate immune cells, increased TNF-α is a major regulator of the early pro-inflammatory response to viral infection in patients with COVID-19. The chemokine MCP-1 levels were increased in post-COVID-19 female patients and maintained a positive correlation with the CRP levels in the post-COVID-19 patients. After recovery from COVID-19, the levels of circulating pro-inflammatory IL-6 decreased after 7 months and, moreover, 12 months of follow-up [32]. We detected increased IL-6 levels in COVID-19 male patients at diagnosis, which remained elevated in the post-COVID-19 patients for up to 5 months. Pro-inflammatory IL-6 participates in the hyperinflammatory response during COVID-19 infection and has a prolonged normalization in post-COVID-19 patients. The serum levels of IFN-γ, TGF-β, and IL-8 are increased in patients with COVID-19 [9]. We presented a temporary reduction of IFN-γ levels in post-COVID-19 female patients. We also showed increased levels of the chemokine IL-8 in our COVID-19 patients. Significantly higher levels of the anti-inflammatory cytokine IL-10 were found in COVID-19 patients, while the levels of IL-10 progressively increased, and those of testosterone progressively decreased with disease severity [33]. The IL-10 and testosterone levels normalized after 7 months [33], while in our study, they normalized after 5 and 2.5 months, respectively. Increased anti-inflammatory IL-10 responded to hyperinflammation during SARS-CoV-2 infection and normalized during recovery in post-COVID-19 patients. Vaccination did not influence the levels of cytokine, chemokines, and hormones in our COVID-19 patients, except for a greater IL-6 response associated with vaccination, indicating an activated immune system.

Estrogen enhances the antiviral defenses and immune activity and downregulates the ACE2 receptor in several tissues [34]. Previous studies reported that estradiol concentrations were not associated with the severity of COVID-19 in men and women [24], while the testosterone levels were similar in female patients regardless of disease severity [22]. This is consistent with our study showing stable estradiol levels in COVID-19 patients, which increased only in post-COVID-19 female patients. However, other studies showed that significantly higher levels of estradiol were found in COVID-19 patients [33]. Positively correlated with IFN-γ, elevated estradiol was associated with a higher probability of severe outcome and death in COVID-19 patients [24,25].

COVID-19 patients showed severe glutathione deficiency, increased oxidative stress, and oxidant damage that worsened with age [35]. SARS-CoV-2 infection is associated with an imbalance between exacerbated oxidative stress and reduced nitric oxide bioavailability proportional to the disease severity [36]. We demonstrated that supraphysiological vs. physiological concentrations of DHT augmented ROS in neutrophils of the COVID-19 patients in vitro, while estradiol had a minor effect. The amount of plasma MPO–DNA complexes, soluble and cellular factors that trigger NETs, is increased in patients with severe COVID-19, and pulmonary autopsies confirmed the presence of NET-containing microthrombi in deceased COVID-19 patients [37]. ICU COVID-19 patients showed higher levels of NET markers (MPO, MPO-DNA, and histone H3) and cell-free DNA that were strongly correlated with CRP, D-dimer, and LDH, while the expression of histone H3 was associated with thromboembolic events [38]. We confirmed elevated plasma levels of NET markers (MPO and histone H3) in our COVID-19 patients on hospital admission. The treatment of neutrophils with COVID-19 platelet-rich plasma generated tissue factor-bearing NETs that induced thrombotic activity in endothelial cells [39].

Endothelial dysfunction has been implicated in thrombotic microangiopathy in patients with severe COVID-19, with increased circulating endothelial cells and plasma levels of soluble ICAM-1, VCAM-1, and P-selectin [5,36,40]. Hypercoagulability, endothelial dysfunction (increased ICAM-1 levels), and inflammation (increased IL-6 levels) can still be detected in some patients approximately 1 year after recovery from COVID-19 [41]. We presented increased and sustainable ICAM-1 plasma levels in COVID-19 and post-COVID-19 patients, which were greater in females. Furthermore, the presented study revealed increased and sustained VCAM-1 plasma levels in post-COVID-19 male patients. Higher levels of soluble P-selectin and E-selectin were detected in COVID-19 patients as predictors of thrombosis, while the E-selectin levels predicted admission to ICU [42]. The P-selectin plasma levels were reduced in the presented COVID-19 and post-COVID-19 female patients vs. the corresponding male patients. We detected increased E-selectin plasma levels in the post-COVID-19 female patients.

In our vitro studies, we used androgen receptor blockade to demonstrate the androgen dependence of neutrophil activation. However, a limitation of this study is the lack of a possible explanation for the molecular mechanisms driving the sexually dimorphic immune response in COVID-19 patients. Studies of human immune cells and mice have shown an immunosuppressive impact of androgens mostly associated with a decreased and/or supported expression of pro-inflammatory and anti-inflammatory mediators [16,43]. Another limitation of our study is the reduction in the number of COVID-19 patients in the extended clinical follow-up. The availability of clinical data is limited by ethical and clinical constraints. The limitations of this single-center study can be overcome by multicenter studies or meta-analyses.

## 5. Conclusions

In addition to TNF-α, the levels of the adhesion molecules ICAM-1 and E-selectin were greatly increased, especially in female COVID-19 patients. The adhesion molecules P-selectin and VCAM-1 were more upregulated in male patients, while the IL-6 levels were generally increased in our COVID-19 and/or post-COVID-19 patients. Vaccination increased the hyperinflammatory response linked to IL-6 in the COVID-19 patients. The NETs and ROS were increased in the plasma and neutrophils of the COVID-19 patients, with the former stimulated by DHT in male neutrophils. Supported by previous reports, this comprehensive study with correlation analyses clearly demonstrated sex steroid hormone-related differences in oxidative stress, adhesion molecule expression, and immune response to SARS-CoV-2 infection in COVID-19 patients. This sex-based approach offers a substantial foundation for the specific management of male patients with COVID-19, considering the potential protective role of testosterone.

## Figures and Tables

**Table 1:** The biochemical parameters of COVID-19 patients at diagnosis and clinical follow-up.

COVID-19	Sex		INR	PTS	UREA mmol/L	CRT μmol/L	CK U/L	D- DIMER mg/L	CRP mg/L	AST U/L	ALT U/L	GGT U/L	FBN g/L	LDH U/L
Diagnosis	M	AV	1.20	12.98	6.70	93.19	380.08	21.38	90.56	59.80	74.06	65.42	5.91	318.85
		SD	1.07	1.03	2.64	33.88	589.24	146.6	68.46	50.97	56.76	58.83	2.12	138.45
		M *	1.08	13	6.05	95	157	0.75	82.9	42	55.5	50	5.4	282
	F	AV	1.11	13.00	6.37	81.20	122.00	2.18	63.62	42.80	45.08	43.72	5.13	268.49
		SD	0.28	1.58	4.79	50.03	104.77	7.65	48.49	36.42	27.84	27.05	1.67	96.01
		M *	1.05	12.7	5.4	74.5	77	0.72	47.5	31	37	36	4.7	258.5
2.5 months	M	AV	2.56 ^3^	11.95 ^3^	6.91	93.75 ^3^	131.5 ^3^	0.56 ^3^	5.40 ^3^	23.15 ^3^	48.61 ^3^	37.15 ^3^	3.17 ^3^	179.18 ^3^
		SD	12.32	1.06	9.30	26.19	153.62	0.65	11.78	11.98	35.17	21.50	1.20	47.60
		M *	0.99	11.8	5.85	91	104	0.36	1.2	20.5	35	31	2.9	172
	F	AV	0.99 ^3^	12.25 ^3^	5.51	70.40	89.46	0.59 ^1^	4.81 ^3^	21.10 ^3^	34.07	33.52 ^1^	3.87 ^3^	173.75 ^3^
		SD	0.08	2.05	1.79	17.13	61.57	0.38	6.46	8.75	13.90	18.13	1.40	48.39
		M *	0.97	11.70	5.15	70.50	70.50	0.49	1.70	18	31	28	3.45	170
5 months	M	AV	0.98 ^3^	11.71 ^3^	6.40	92.10	163.44	0.55 ^3^	6.09 ^3^	25.71 ^3^	47.88 ^3^	32.28 ^3^	2.96 ^3^	166.82 ^3^
		SD	0.07	0.72	1.98	27.23	153.89	0.78	14.69	20.06	43.22	11.70	1.11	32.02
		M *	0.97	11.6	6	87	116.5	0.33	1.6	21	33	29	2.7	159
	F	AV	1.03 ^3^	12.36 ^3^	5.85	60.27 ^2^	95.50	3.84 ^1^	5.38 ^3^	17.96 ^3^	29.67 ^1^	26.50 ^3^	3.77 ^3^	153.24 ^3^
		SD	0.26	2.93	1.72	19.43	50.59	14.88	12.05	6.37	11.03	17.84	1.61	42.40
		M *	0.97	11.7	5.55	60	87	0.425	2.25	17	26	19.5	3.2	160
Normal range			0.8–1.1	11–13.5	2.5–7.5	59–104	0–200	0.5	0–3	0–37	14–63	0–55	1.8–3.5	85–227

^1^*p* < 0.05; ^2^ *p* < 0.01; ^3^ *p* <0.001 vs. values at diagnosis. CRT—Creatinine; FBN—Fibrinogen; PTS—prothrombin time (s); * Median (M).

**Table 2:** Significant correlation of the levels of inflammatory cytokines, chemokines, cell adhesion factors, and sex hormones with biochemical parameters in the examined COVID-19 and post-COVID-19 patients.

*p* Values	Sex	INR	PTS	Urea	Creatinine	CK	D-dimer	CRP	AST	ALT	GGT	Fibrinogen	LDH	Vaccine	CO	Co-M
E-SELECTIN	F		**0.015 ^2^**			**0.006 ^1^**							**0.048** **0.001 ^2^**	0.021		
	M								0.009 ^2^					0.025 ^2^		
	T								**0.033**		**0.048**		**0.023**	0.029		0.026 0.021 ^1^
P- SELECTIN	F				0.000 ^1^				0.049	0.005	0.044	0.005				
	M													**0.040 ^2^**		**0.024 ^2^**
	T					0.022 ^2^				0.031						
VCAM-1	F									0.046 ^1^			**0.030 ^2^**			**0.037**
	M				0.020 ^2^						0.037 ^2^	0.048 ^2^		0.001 ^1^	0.022 ^1^	
	T				0.007 ^2^				0.007 ^2^	0.008 ^2^	0.023 ^2^				0.008 ^1^	
ICAM-1	F													0.043 ^1^		
	M		0.032											0.024 ^1^		
	T				**0.005 ^1^**											
ESTRADIOL	F	0.013 ^1^	0.009 ^1^	0.011 ^1^	0.016 ^1^	0.030 ^1^										
	M			0.002 ^2^												0.006 **^1^** 0.018 ^2^
	T					0.046 ^1^										
DHT	F					0.017							0.025		0.028	
	M				**0.036**											0.028
	T					0.004 0.018 ^2^	**0.013 ^2^**	**0.049 ^2^**						0.005 ^1^		
SHBG	F	**0.046**			**0.003 ^1^**	**0.024**							**0.037 ^1^**			
	M				**0.035**							**0.011 ^2^**				
	T					**0.001** **0.030 ^1^**					**0.048 ^1^**					0.003
INF-γ	F			**0.021 ^2^**	0.001 ^1^							0.03 ^1^				
	M					**0.015**	**0.006 ^2^**							**0.041 ^1^** **0.040 ^2^**		
	T				0.001 ^1^	**0.015**										0.014
TNF-α	F										**0.028 ^1^**					
	T									**0.016 ^1^**						
IL-10	F			0.012 ^1^			0.011 ^2^							0.037 ^2^		
	T									**0.028 ^2^**						
IL-6	F											0.04				
	M							**0.035 ^2^**								
	T					0.024 ^2^		**0.011 ^2^**					0.004 ^2^			
IL-1β	F														0.003 ^1^	
	M					**0.006 ^1^**										0.027 ^1^
	T					**0.004 ^1^**										
MCP-1	F											0.027				
	M	0.022 ^1^					0.013 ^2^									
	T	0.003 ^1^					0.004 ^2^	0.019 ^2^								
TGF-β	M											**0.016**				
	T	0.021 ^2^	0.012 ^2^												0.042 ^1^	**0.046**
IL-8	F											**0.032**				
	M			**0.029**											**0.047 ^2^**	
	T			**0.029** ^2^											0.007	0.036

^1^ After first control (2.5 months); ^2^ After second control (5 months) of hospital admission; No superscript (at diagnosis). Bolded values have a negative correlation, while the others have a positive correlation. F—female; M—male; T—total; CO—clinical outcome; Co-M—comorbidities.

## Data Availability

The data are not publicly available due to privacy and ethical restrictions.

## Supplementary Materials

The following supporting information can be downloaded at https://www.mdpi.com/article/10.3390/vaccines11101560/s1. Table S1: Number and sex of COVID-19/post-COVID-19 patients per observed cytokines; Table S2: ELISA kits of applied cytokines; Table S3: Concentration values for all examined sex hormones, cytokine/chemokines and adhesion molecules in COVID-19 and post-COVID-19 patients; Table S4: The complete blood count test of COVID-19 patients at diagnosis and clinical follow-up; Table S5: Spearman’s correlation of biochemical parameters with the levels of inflammatory cytokines, chemokines, cell adhesion factors and sex hormones in the examined COVID-19 and post-COVID-19 male and female patients.
