# Supplementary material for: Sex Differences and Cytokine Profiles among Patients Hospitalized for COVID-19 and during Their Recovery: The Predominance of Adhesion Molecules in Females and Oxidative Stress in Males
Source: Vaccines (Basel). 2023 Oct 3;11(10):1560. doi: 10.3390/vaccines11101560 (PMC10610714; doi:10.3390/vaccines11101560)
Supplement: Supplementary file 1 [file vaccines-11-01560-s001.zip › vaccines-2525066-supplementary.pdf]

Table S1. Number and sex of healthy donors and COVID-19 /post-COVID-19 patients per observed cytokines.

Cytokines	E-SELECTIN	MCP-1	IL-10	P-SELECTIN	ESTRADIOL	TGF-β	SHBG	INF-γ	TNF-α	VCAM-1	ICAM-1	IL-8	IL-6	IL-1β	DHT	Testosterone	Free testosterone
**Total**							**At diagnosis**										
№ of patients	71	78	47	49	81	78	70	54	50	55	54	55	56	55	91	13	13
**Female COVID-19 patients**																	
№ of patients	19	20	20	21	27	20	24	18	18	18	18	19	18	18	27	6	6
**Male COVID-19 patients**																	
№ of patients	52	58	27	28	54	58	46	36	32	36	36	36	38	36	64	7	7
**Total**							**2.5 months after hospital admission**										
№ of patients	79	55	55	60	44	58	38	66	41	41	41	66	67	65	69	13	13
**Female post-COVID-19 patients**																	
№ of patients	27	16	28	28	17	17	15	27	15	15	15	27	27	26	17	6	6
**Male post-COVID-19 patients**																	
№ of patients	52	39	27	32	27	41	23	39	26	26	26	39	40	39	52	7	7
**Total**							**5 months after hospital admission**										
№ of patients	48	41	42	35	35	39	22	35	22	22	22	34	34	35	54	14	14
**Female post-COVID-19 patients**																	
№ of patients	16	13	22	15	15	13	10	15	10	10	9	15	15	15	15	6	6
**Male post-COVID-19 patients**																	
№ of patients	32	28	20	20	20	26	12	20	12	12	13	19	19	20	39	8	8
**Total**							**healthy donors**										
№ of donors	17	18	18	25	8	19	8	16	19	8	8	22	21	18	8	10	10
**Female healthy donors**																	
№ of donors	10	10	10	14	4	10	4	9	11	4	4	12	11	10	4	5	5
**Male healthy donors**																	
№ of donors	7	8	8	11	4	9	4	7	8	4	4	10	10	8	4	5	5

Table S2. ELISA kits of applied cytokines.

ELISA Assay	Units	Cat. No.	Manufacturer
**Dihydrotestosterone (DHT)**	pg/ml	E-EL-0031	Elabsciences Biotechnology Inc., Houston, Tx, USA
**E2 (Estradiol)**	pg/ml	E-OSEL-H0005	Elabsciences Biotechnology Inc.
**Sex hormone binding globulin (SHBG)**	pmol/ml	E-EL-H6100	Elabsciences Biotechnology Inc.
**Interleukin – 6 (IL-6)**	pg/ml	E-EL-H0102	Elabsciences Biotechnology Inc.
**Interleukin – 10 (IL-10)**	pg/ml	E-EL-H0103	Elabsciences Biotechnology Inc.
**Interleukin – 1β (IL-1β)**	pg/ml	E-EL-H0149	Elabsciences Biotechnology Inc.
**Interleukin – 8 (IL-8)**	pg/ml	E-EL-H6008	Elabsciences Biotechnology Inc.
**Interferon gamma (IFN-γ)**	µg/ml	E-EL-H0108	Elabsciences Biotechnology Inc.
**Transforming growth factor beta (TGF-β)**	ng/ml	E-EL-0162	Elabsciences Biotechnology Inc.
**Tumor necrosis factor alpha (TNF-α)**	pg/ml	E-EL-H0109	Elabsciences Biotechnology Inc.
**Monocyte chemoattractant protein-1 (MCP-1)**	pg/ml	E-EL-H6005	Elabsciences Biotechnology Inc.
**Intercellular Adhesion Molecule 1 (ICAM-1)**	pg/ml	E-EL-H6114	Elabsciences Biotechnology Inc.
**Vascular Cell Adhesion Molecule (VCAM-1)**	µg/ml	E-EL-H5587	Elabsciences Biotechnology Inc.
**P-selectin**	ng/ml	E-EL-H0917	Elabsciences Biotechnology Inc.
**E-selectin**	pg/ml	E-EL-H0876	Elabsciences Biotechnology Inc.

Table S3. Concentration values for all examined sex hormones, cytokine/chemokines and adhesion molecules in healthy donors, COVID-19 and post-COVID-19 patients.

	Sex		Age	TT nmol/l	Free TT pg/ml	DHT pg/ml	SHBG pmol/ml	EST pg/ml	IL8 pg/ml	IL6 pg/ml	TNFα pg/ml	INFγ µg/ml	IL10 pg/ml	ICAM1 µg/ml	VCAM1 µg/ml	E-sel pg/ml	P-sel pg/ml	IL1β pg/ml	TGFβ ng/ml	MCP1 pg/ml
Diagnosis	Male	AV	54.91	6.27	8.31	139.35	10.14	11.39	16.33	66.83	10.84	11.05	13.17	9.39	57.72	1430.24	8.00	9.75	3.83	586.59
		SD	14.38	2.58	4.44	87.63	6.50	2.42	7.76	46.65	6.71	2.41	14.56	3.51	25.76	586.06	1.25	2.11	0.98	383.16
		M*	55	6.18	8.10	122.50	7.56	11.42	14.44	55.79	9.49	10.08	9.28	8.06	55.28	1433.57	7.84	9.74	3.93	455.71
	Female	AV	61.29	0.97	1.82	111.54	22.20	10.96	19.71	49.05	41.98	12.35	17.00	12.09	64.78	1740.26	6.77	11.4	3.67	612.93
		SD	13.87	0.28	0.22	55.88	11.72	2.32	7.48	26.33	40.91	4.96	31.40	5.20	33.37	1187.39	1.11	3.58	0.98	402.20
		M*	62	0.98	1.80	103.60	22.18	11.42	17.94	64.11	21.84	11.82	8.17	9.72	57.81	1661.30	7.10	10.89	3.80	567.79
2.5 months	Male	AV	55.85	13.81	19.25	96.61	6.72	12.62	17.17	36.66	9.22	11.15	5.71	18.58	75.42	1766.87	6.50	9.63	4.06	563.52
		SD	14.30	4.31	6.87	66.48	8.54	2.78	7.31	46.35	0.91	1.49	4.71	3.59	34.28	792.66	2.03	0.82	2.70	270.44
		M*	55.5	13.55	19.75	105.00	3.09	12.70	15.24	26.87	9.35	11.44	4.28	18.73	73.52	1524.17	6.43	9.79	3.66	528.71
	Female	AV	61.28	0.84	2.00	81.86	27.17	12.73	14.69	48.82	9.19	5.36	7.51	23.13	54.48	2761.94	5.51	9.49	3.70	899.58
		SD	12.57	0.58	0.49	39.31	9.23	2.68	4.80	49.42	0.43	3.70	11.98	5.03	26.75	992.75	1.44	0.83	2.48	481.34
		M*	62	0.84	2.00	101.16	26.21	12.54	15.15	21.72	9.26	2.99	4.72	23.28	44.12	2781.67	5.20	9.78	2.97	789.26
5 months	Male	AV	54.54	14.47	26.46	129.78	6.24	12.26	15.02	32.53	8.79	10.73	4.77	18.12	71.23	2118.53	6.56	9.49	3.88	575.00
		SD	17.10	5.17	9.84	78.64	6.54	3.15	5.25	33.20	1.15	2.01	2.02	3.96	39.31	1100.43	2.44	0.89	2.88	388.97
		M*	56.5	14.79	22.40	113.11	4.97	11.96	13.85	25.29	9.14	11.59	4.53	18.50	57.62	1770.88	6.08	9.79	2.79	456.00
	Female	AV	61.36	0.56	1.63	76.52	21.51	13.90	17.00	18.70	9.23	10.89	4.68	21.31	34.29	2829.51	5.42	9.81	3.52	528.71
		SD	10.65	0.30	0.29	36.29	7.51	2.25	5.22	21.27	0.32	1.51	2.02	4.29	36.47	1013.05	1.57	1.18	2.41	182.02
		M*	62	0.58	1.70	85.23	23.79	13.59	16.81	9.58	9.29	11.61	4.72	21.44	23.94	2627.67	4.88	9.79	2.75	599.33
Healthy donors	Male	AV	41.18	19.53	29.84	169.76	3.20	12.28	8.39	12.25	5.90	8.45	6.97	7.38	36.48	1927.14	5.25	15.18	9.98	449.25
		SD	12.39	9.09	7.61	68.13	0.95	1.22	2.77	3.85	0.93	0.51	7.18	1.66	6.84	617.61	1.85	17.17	5.26	194.25
		M*	41	17.60	29.48	170.24	2.82	12.23	7.82	13.66	5.83	8.37	5.00	7.42	34.85	2049	4.00	9.56	11.58	478.86
	Female	AV	35.15	1.10	2.13	62.38	14.11	11.48	10.86	20.44	7.49	9.01	4.41	7.13	49.98	1514.40	5.40	9.55	9.29	313.80
		SD	10.10	0.37	0.60	26.62	9.50	0.94	4.68	19.93	2.85	0.91	1.20	0.81	13.24	787.98	1.94	1.68	3.65	112.11
		M*	35	1.17	1.99	56.08	10.24	11.23	9.26	13.95	6.07	9.13	4.37	7.07	48.90	1520.17	5.64	8.97	9.96	289.50

TT – testosterone, EST – Estradiol, sel – selectin, M* - Median

Table S4. The complete blood count test of COVID-19 patients at diagnosis and clinical follow-up.

COVID19	Sex		RBC $x10^{12}/L$	HGB g/L	HCT L/L	MCV fL	MCH pg	MCHC g/L	RDW %	WBC $x10^{9}/L$	LYMPH %	NEUT %	MONO %	MPV fL	PLT $x10^{9}/L$
Diagnosis	Male	AV	4.79	143.79	0.42	87.87	29.94	340.79	12.70	7.31	17.72	73.88	7.70	9.99	219.74
		SD	0.57	13.03	0.04	4.24	1.55	8.75	1.20	3.52	9.77	11.91	4.92	0.68	106.39
		M*	4.84	145.00	0.43	87.90	29.90	340.00	12.40	6.50	15.90	73.9	6.4	10.05	194
	Female	AV	4.40	130.10	0.61	87.98	29.65	336.85	13.07	6.01	21.67	70.60	6.75	9.96	216.54
		SD	0.49	13.06	1.42	3.72	1.57	8.57	1.78	2.65	9.48	10.77	2.87	0.96	86.74
		M*	4.43	130	0.383	87.65	29.7	336.5	12.8	5.55	19.75	72.15	6.35	9.75	212.5
2.5 months	Male	AV	4.83	145.83	0.43	89.38[1]	30.34	339.4[2]	13.3[3]	7.06	29.7[3]	57.25[3]	8.74[2]	11.26	255.64[2]
		SD	0.50	11.64	0.03	4.60	1.70	10.55	1.18	2.46	9.56	10.65	2.27	10.92	149.00
		M*	4.86	146.00	0.43	89.05	30.15	338.00	13.15	6.60	30.05	56.65	8.70	9.85	233.50
	Female	AV	4.44	133.19	0.40	90.36[1]	30.09	332.9[1]	13.33	6.22	32.74[3]	55.91[3]	8.19[1]	10.03	284.19[3]
		SD	0.44	9.89	0.03	4.05	1.46	7.08	1.06	1.65	7.10	8.06	1.76	0.84	96.33
		M*	4.55	135.00	0.40	90.70	30.20	332.00	13.10	6.50	32.90	56.00	8.00	10.10	264.00
5 months	Male	AV	5.03[1]	150.42[2]	0.44[2]	88.73	30.03	338.19	12.95	6.82	31.84[3]	56.17[3]	8.91[2]	9.92[3]	232.7[3]
		SD	0.51	11.63	0.03	4.16	1.70	10.00	1.84	1.41	9.38	9.54	2.51	0.83	56.67
		M*	5.04	150.00	0.45	88.30	29.85	338.00	12.75	6.65	30.65	57.75	9.05	9.70	232.7
	Female	AV	4.65[1]	137.72[1]	0.41	88.54	29.62	334.56	13.00	6.53	31.13[3]	59.02[3]	7.11	9.96[3]	257.1[3]
		SD	0.39	11.57	0.03	3.39	1.40	7.82	0.98	2.30	7.28	8.51	1.78	0.85	62.84
		M*	4.73	137.50	0.41	88.40	29.60	334.50	12.75	6.10	31.80	57.25	7.20	9.75	257.12
Normal range			4.34-5.72	138-175	0.415-0.53	80-100	27.4-33.9	320-360	12-15.5	3.4-9.7	20-46	44-72	2-12	6.8-10.4	158-424

[1]$p<0.05$; [2]$p<0.01$; [3]$p<0.001$ vs. values at diagnosis, M* - Median

Table S5. Spearman correlation of biochemical parameters with the levels of inflammatory cytokines, chemokines, cell adhesion factors and sex hormones in the examined COVID-19 and post-COVID-19 male and female patients.

Spearman's r	Sex	INR	PTS	UREA	Creatinine	CK	D-dimer	CRP	AST	ALT	GGT	Fibrinogen	LDH	Vaccine	CO	Co-M
E-SELEKTIN	F		-.758[2]			-.762[1]							-.490[2]<br>-.875[2]	.563		
	M								.543[2]					.407[2]		
	T					-.246			-.314		-.29		-.301	.267		.272<br>.274[1]
P-SELEKTIN	F				.586[1]				.486	.655	.514	-.627				
	M													-.462[2]		-.503[2]
	T					.523[2]				.326						
VCAM-1	F									.567[1]			-.731[2]			.529
	M				.639[2]						.762[2]	.383[2]		.606[1]	.457[1]	
	T				.569[2]				.569[2]	.573[2]	.589[2]				.417[1]	
ICAM-1	F													-.554[1]		
	M		.576											.45[1]		
	T				-.451[1]											
ESTRADIOL	F	.634[1]	.659[1]	.672[1]	.620[1]	.636[1]										
	M			.754[2]												.526[1]<br>.522[2]
	T					.350[1]										
DHT	F					.473							.447		.439	
	M				-.269											.281
	T					.309<br>.365[2]	-.349[2]	-.283[2]						.349[1]		
SHBG	F	-.545			-.496[1]	-.489							-.568[1]			
	M				-.311							-.778[2]				
	T					-.386<br>-.396[1]					-.472[1]					.362
INF-γ	F			-.762[2]	.535[1]							.486[1]	.			
	M					-.407	-.626[2]							-.338[1]<br>-.451[2]		
	T				.405[1]	-.337										.34
TNF-α	F										-.74[1]					
	T									-.398[1]						
IL-10	F			.683[1]			.857[2]							.596[2]		
	T									-.518[2]						

IL-6	F							.518			
	M						-.594[2]				
	T				.529[2]		-.553[2]		.489[2]		
IL-1β	F									.571[1]	
	M				-.546[1]						.364[1]
	T				-.463[1]						
MCP-1	F							.604			
	M	.390[1]				0.497[2]					
	T	.425[1]				0.483[2]	.389[2]				
TGF-β	M							-.466			
	T	.40[2]	.453[2]							.288[1]	-.243
IL-8	F							-.52			
	M			-.381						-.449[2]	
	T			-.381[2]						.368	.289

[1]After first control (2.5 months); [2]After second control (5 months) of hospital admission; No superscript (at diagnosis). F-female, M-male, T-total. CO – Clinical outcome, Co-M – Comorbidities.
